# Application of Improved CycleGAN in Laser-Visible Face Image Translation

**DOI:** 10.3390/s22114057

**Published:** 2022-05-27

**Authors:** Mingyu Qin, Youchen Fan, Huichao Guo, Mingqian Wang

**Affiliations:** 1 Graduate School, Department of Electronic and Optical Engineering, Space Engineering University, Beijing 101416, China; 15544029418m@sina.cn (M.Q.); mqwang0302@163.com (M.W.); 2 School of Space Information, Space Engineering University, Beijing 101416, China; 3 Department of Electronic and Optical Engineering, Space Engineering University, Beijing 101416, China; guohuichaoo@163.com

**Keywords:** CycleGAN, least squares method, identity loss, RRDB module

## Abstract

CycleGAN is widely used in various image translations, such as thermal-infrared–visible-image translation, near-infrared–visible-image translation, and shortwave-infrared–visible-image translation. However, most image translations are used for infrared-to-visible translation, and the wide application of laser imaging has an increasingly strong demand for laser–visible image translation. In addition, the current image translation is mainly aimed at frontal face images, which cannot be effectively utilized to translate faces at a certain angle. In this paper, we construct a laser-visible face mapping dataset; in case of the gradient dispersion of the objective function of the original adversarial loss, the least squares loss function is used to replace the cross-entropy loss function and an identity loss function is added to strengthen the network constraints on the generator. The experimental results indicate that the SSIM value of the improved model increases by 1.25%, 8%, 0, 8%, the PSNR value is not much different, and the FID value decreases by 11.22, 12.85, 43.37 and 72.19, respectively, compared with the CycleGAN, Pix2pix, U-GAN-IT and StarGAN models. In the profile image translation, in view of the poor extraction effect of CycleGAN’s original feature extraction module ResNet, the RRDB module is used to replace it based on the first improvement. The experimental results show that, compared with the CycleGAN, Pix2pix, U-GAN-IT, StarGAN and the first improved model, the SSIM value of the improved model increased by 3.75%, 10.67%, 2.47%, 10.67% and 2.47%, respectively; the PSNR value increased by 1.02, 2.74, 0.32, 0.66 and 0.02, respectively; the FID value reduced by 26.32, 27.95, 58.47, 87.29 and 15.1, respectively. Subjectively, the contour features and facial features were better conserved.

## 1. Introduction

**Figure 1:**
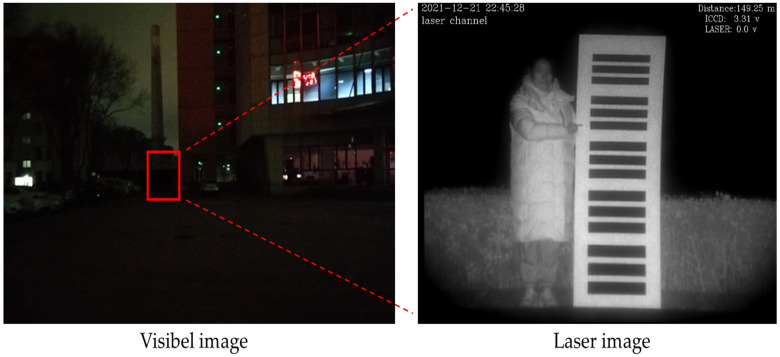
Visible image and laser image.

Most of the monitoring equipment is a visible imaging system, but the visible imaging system is affected by factors such as ambient light and target distance, resulting in lower imaging quality. Laser active imaging uses lasers as the light source for active imaging systems. Compared with visible light imaging, this has advantages such as imaging in dark conditions, a strong anti-interference ability and a long imaging distance, and is widely used in security monitoring and other aspects [1]. As shown in Figure 1, the position of the red box in the visible image is the target in the laser image and the target in the visible image under the same conditions is invisible to the human eye. However, the laser image is gray and lacks color information, which is not conducive to human observation and recognition. By combining the advantages of the visible imaging system in the daytime and the advantages of the range-gated imaging equipment in the night, the effective operation of the security and monitoring equipment can be realized throughout the day. However, due to the differences in spectral characteristics, the laser image is quite different from the visible image. When observing the laser target, the recognition accuracy and reliability of the laser face is low [2]. This hinders the practical application of range-gated imaging equipment. In this paper, computer vision and deep learning is used to translate the laser face image into a visible face image that is more suitable for human observation, so that the monitoring personnel can obtain the facial information of the target person in the monitoring video, and determine the identity of the target.

With the rapid development of computer vision and deep learning, deep neural networks play an increasingly important role in the field of image processing. Image translation is an important branch in the field of image processing, and can convert images in different domains. Goodfellow et al. [3] proposed a generative adversarial model in 2014, an unsupervised algorithm framework based on the idea of a zero-sum game, where the generative network and the discriminant network play against each other and finally reach the Nash equilibrium. However, the Nash equilibrium of the GAN network is not easy to achieve, and the model training is unstable, causing the model to collapse. Gatys et al. [4] proposed a style transfer algorithm based on a neural network in 2015, using the VGG network to extract a feature map of the network, using the deep feature map as the content feature and the shallow feature map as the style feature, and recombining the content feature and style feature for a new image. However, this method cannot be converted in batches; each image translation needs to be reiterated, and the training is time-consuming. Isola et al. [5] proposed Pix2pix in 2016. Unlike GAN, the original generator inputs random vectors and outputs images, and the discriminator inputs images and discriminates between right and wrong. While the Pix2pix generator inputs images and outputs fake images, the discriminator inputs two images to distinguish between real samples and generated samples. However, Pix2pix requires matching images in the two domains, and the dataset is not readily available. Zhu et al. [6] proposed CycleGAN in 2017, creatively proposed the idea of a cycle, reconstructed the input image through two different generators, and obtained excellent results without matching data. Yunjey et al. [7] proposed StarGAN in 2018. Unlike Pix2pix and CycleGAN, which can only be used to solve image translation from one domain to another, StarGAN uses a generative model to solve the problem of image translation from one domain to multiple domains. Junho et al. [8] proposed U-GAN-IT in 2019, which introduced a new attention mechanism to make the generator pay more attention to more important regions. This is more suitable for the field of image translation with large changes in image texture and shape. In 2020, Hu Linmiao et al. [9] proposed a shortwave-infrared–visible-face-image translation based on generative adversarial networks. Taking advantage of the similarities between the Y-channel information of the visible image and the shortwave infrared image, the overall loss function and the Y-channel generation and reconstruction loss function are introduced to the CycleGAN, so that the translated image can obtain color information while losing as little structure and texture as possible. Jingtao et al. [10] proposed a dense generative adversarial network for near-infrared face image colorization in 2021. The generator combines the advantages of DenseNet [11] and Unet [12], enabling the network to extract deeper information.

For heterogeneous face image translation, most existing networks aimed towards frontal face image translation, and the research in the field of profile image translation is insufficient. Most existing face datasets are visible–visible or infrared–visible. For laser–visible-face translation, there is no well-matched and publicly available laser face and corresponding visible face dataset at present.

This article compares CycleGAN, Pix2pix, U-GAN-IT and StarGAN models, and finally chooses to improve CycleGAN. Focusing on the problems of blurred images and inconspicuous facial features in the laser–visible-face translation of CycleGAN, the objective function of the adversarial loss function is improved, and the identity loss function is introduced. Looking at the problem of incorrect mapping in side-face translation, the feature extraction part of the generator is modified to the RRDB module. For the face translation results, the verification is carried out by combining subjective vision and objective quantification.

## 2. Dataset Acquisition and Processing

### 2.1. Data Acquisition

To study the translation from laser face images to visible face images, a laser–visible-face-image dataset was collected and established. In the field of face translation, this can be divided into supervised learning and unsupervised learning. The two supervised learning datasets need to strictly correspond, such as the pose of the collection target and the number of targets in the two domains. Datasets for unsupervised learning do not need to strictly correspond. According to actual needs, when collecting data, the laser image acquisition equipment and the visible image acquisition equipment must have a certain synchronization; that is, the acquisition location, angle and distance from the target should be as close as possible.

The laser image acquisition equipment is a range-gated imaging instrument developed in the laboratory, with two modes: active imaging and passive imaging. In this experiment, the active imaging mode was used to collect laser images. The active imaging mode overcomes backscattering, shields signals other than the target signal, and can acquire high-resolution images. The instrument has a built-in, enhanced ICCD camera, which was used for laser imaging in the near-infrared band. The shutter time is fixed at 80 ns, and the brightness gain can reach 10,000 cd/m^2^/lx. The pixel of the camera is 576 × 768, and the response wavelength is 400–900 nm. The ICCD gain voltage adjustment range is 1.8–5 V, and the initial gain voltage is 1.8 V. The voltage gain was adjusted according to the field conditions during acquisition, and the actual use range was 2.4–2.5 V. The working wavelength of the fiber-coupled pulsed array semiconductor laser module of 860 nm, the peak power of greater than 2 kW, the pulse width of 40 ns and the repetition frequency of 20 kHz were selected as fixed values, which meet the lighting requirements of range-gated imaging equipment. The adjustment voltage range of the laser gain was 0–5 V. The image displayed module displays and saved the collected images with an image resolution of 1024 × 768.

Laser modules used in range-gated imaging equipment can emit strong energy. According to the accessible emission limit and the comparison standard laser hazard classification conditions, the laser hazard level was determined to be Class 4 [13]. Lasers of Class 4 hazard level are more harmful to human eyes when used. Moreover, the laser in the 400 nm–1400 nm band had a convergence effect through the refractive media of the eye, so that the light energy reaching the retina is 10^5^ times higher than the light energy incident to the cornea [14]; therefore, the laser with a wavelength of 860 nm will cause great damage to the retina. To reduce the laser damage to human eyes, the collecting target should close its eyes during acquisition.

The visible collection device was a Canon 60D SLR camera (Tokyo, Japan) with an 18–200 mm zoom lens, a 3-inch 1.04 million pixel TFT LCD screen, and a 22.3 mm × 14.9 mm CMOS sensor. The acquired image resolution was 5184 × 3456.

**Figure 2:**
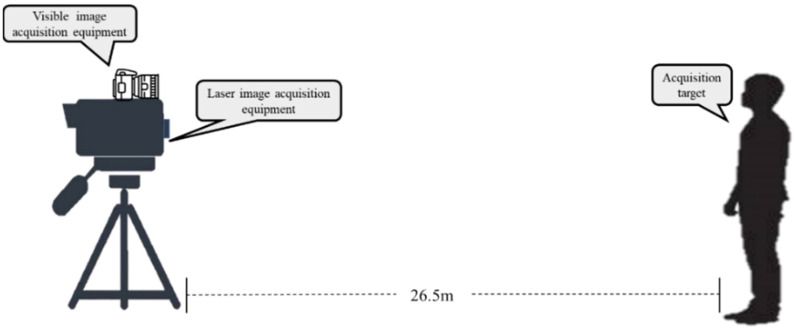
Schematic diagram of the image acquisition scene.

Image acquisition was carried out in a long corridor indoors. A switch was used to control the corridor lights. Visible images were collected under incandescent lamps, and laser images were collected under dark conditions. The collection environment is shown in Figure 2. The visible image collection device was located above the laser image collection device, and the two devices were located directly in front of the collection target, 26.5 m from the collection target. Images of 100 human objects were collected in sequence, including 78 male objects and 22 female objects, and the frontal and 30-degree left-turned face images of the human objects were collected. We tried to ensure that all targets were in the same position during acquisition, and avoided repeatedly adjusting the focal length of the device. To reduce the damage from the laser to the human eyes, the collection target was required to close his eyes during collection.

### 2.2. Data Processing

**Figure 3:**
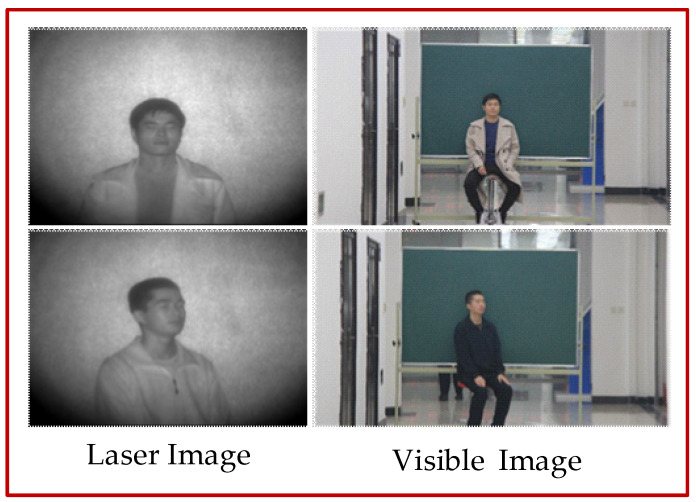
Acquired laser and visible images.

The field of view of the range-gated imaging instrument was much smaller than that of the Canon 60D visible camera. Although the target people were in the middle of the field of view, the size and proportion of the face were different. There was also a lot of noise information in the background of the laser image, and a complex background in the visible image, which are important factors that affect the translation quality. Five steps of face recognition, face alignment, face cropping, face segmentation and image normalization were used for image preprocessing. The collected laser images and visible images are shown in Figure 3; the first column shows the laser image and the second column shows the visible image.

**Figure 4:**
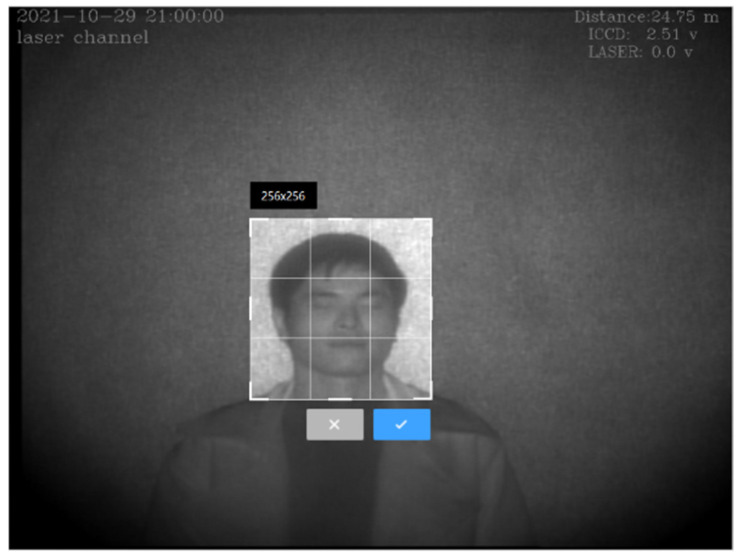
The size of the laser head in the entire image.

Firstly, face detection was performed, using Dilb to identify the face in the original image and extract 68 facial feature-points. When the inclination of the face is different, this increases the difficulty of network training, so the second step was to align the image according to the outer corners of the left and right eyes. The laser face and the visible face occupied a small proportion of the whole image, and the image sizes of the two domains were different. These factors also increase the difficulty of network training; as a result, the third step was to cut out the face part in the image, according to 68 feature points. The background of the laser image and visible light image was cluttered, which is not conducive to the generator learning. The simple background made the outline of the face clear and increased the generator’s ability to pay attention to important information; therefore, the fourth step was to perform face segmentation and fill the cluttered background with white. Finally, as size of the human head in the original laser image is roughly 256 × 256, as shown in Figure 4, if the image becomes high resolution, the image quality will be degraded, which is not conducive to the generator learning the correct mapping relationship. Therefore, the processed image was normalized to 255 × 256. Likewise, visible light images were also normalized to 256 × 256.

**Figure 5:**
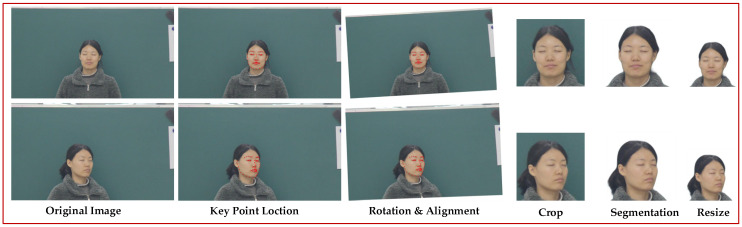
Dataset preprocessing process.

The dataset processing flow is shown in Figure 5. 

In the field of deep learning, too few data can easily lead to poor network learning results. The image of the two domains was flipped to increase the number of images. Finally, the training datasets included 382 laser face images and 382 visible face images.

## 3. Image Translation with Generative Adversarial Network

### 3.1. Analysis of Generative Adversarial Networks Characteristics

Generative adversarial networks have great advantages in image style transfer, image synthesis and image translation [15]. CycleGAN, Pix2pix, U-GAN-IT and StarGAN are all classic image translation algorithms, all of which have shown good results. As an unsupervised algorithm, CycleGAN can complete image translation with the unpaired datasets, especially for translation tasks where image contours have no major changes in two domains [16,17]. As a supervised algorithm, the Pix2pix network requires paired datasets for training. However, there are some differences in the laser’s angle and field of view, as well as visible acquisition device collecting images, meaning that the laser face image does not strictly correspond to the visible face image. As an unsupervised image translation network, U-GAN-IT introduces an attention mechanism and can be applied to image translation with large differences in style and texture. The above models all solve one-to-one problems, but the StarGAN model solves the problem of multi-domain image translation, and one model can solve multiple image translation tasks.

### 3.2. CycleGAN Model

**Figure 6:**
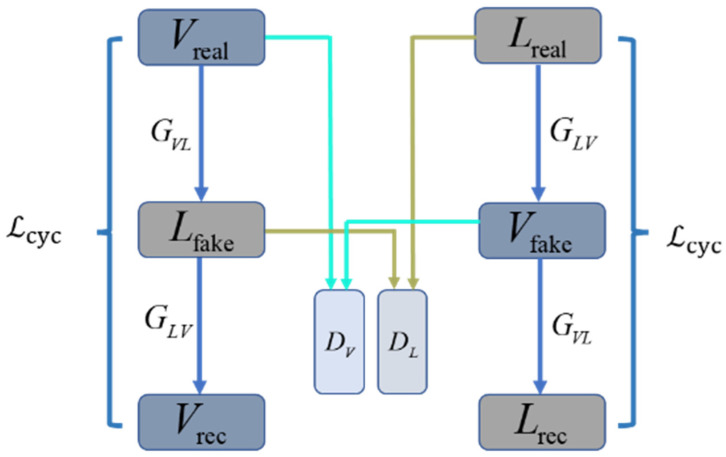
CycleGAN network structure.

CycleGAN consists of two mirrored links, each consisting of two generators and a discriminator, as shown in Figure 6. Taking the visible–laser–visible link as an example, the two generators are $G_{VL}$ and $G_{LV}$, where $G_{VL}$ is the generator of visible-to-laser, and $G_{LV}$ is the generator of laser-to-visible. $V_{real}$ is an image in the visible domain, $V_{rec}$ is a reconstructed visible image, and $L_{fake}$ is a generated fake laser image. The CycleGAN model first input the image $V_{real}$ into the generator $G_{VL}$ to obtain the fake image $L_{fake}$, and then input $L_{fake}$ into the second generator $G_{LV}$ to obtain the reconstructed image $V_{rec}$. Similarly, another link input the image $L_{real}$ to the generator $G_{LV}$ to obtain a fake image $V_{fake}$, and input the image $V_{fake}$ to the second generator $G_{VL}$ of the link to obtain the reconstructed image $L_{rec}$. The discriminator $D_{V}$ discriminates the authenticity of the images $V_{real}$ and $V_{fake}$, and the discriminator $D_{L}$ discriminates the authenticity of the images $L_{real}$ and $L_{fake}$. The CycleGAN introduces the concept of cycle consistency. The image $V_{real}$ should theoretically be infinitely close to the image $V_{rec}$ in distribution, and the image $L_{real}$ should theoretically be infinitely close to the image $L_{rec}$ in distribution.

**Figure 7:**
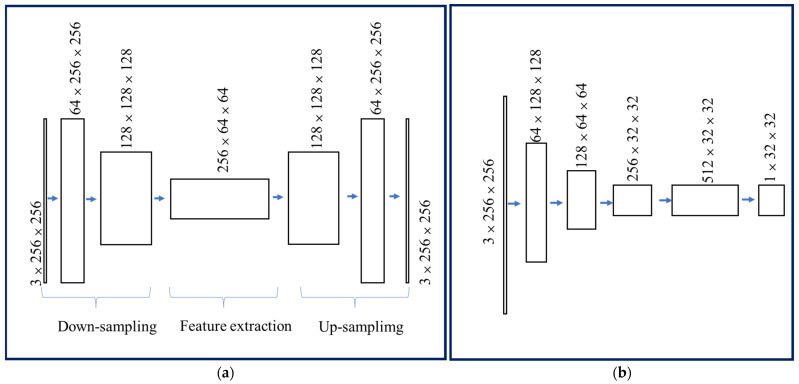
(**a**) Generator structure. (**b**) Discriminator structure.

The generator consists of three modules of down-sampling feature extraction and up-sampling, as shown in Figure 7a. Down-sampling consists of three Conv + IN + ReLU structures. An image of size 3 × 256 × 256 was down-sampled into 256 feature maps of 64 × 64. The feature extraction module consisted of nine residual blocks, and the input and output size was unchanged. Up-sampling consists of two Conv + IN + ReLU and one Conv + Tanh. A 64 × 64 feature map of number 256 obtained an image of size 3 × 256 × 256.

The discriminator network uses the PatchGAN structure. PatchGAN output an N × N matrix, and each element in the matrix represents the discrimination result of a small area in the input image. Compared with ordinary discriminators, PatchGAN has fewer parameters and a more accurate discriminative ability. It consists of four Conv + IN + LeakyReLU and one Conv, and the discriminator is shown in Figure 7b.

The loss function has a great influence on image translation. In CycleGAN, the adversarial loss function and the cycle consistency loss function are used.

Adversarial loss function:

(1) $$L_{GAN}(G_{VL},D_{L},L,V)=E_{l∼\mathrm{pdata}(l)}[\log(D_{L}(l))]+E_{v∼\mathrm{pdata}(v)}[1-\log(D_{L}(G_{VL}(v)))]$$

(2) $$L_{GAN}(G_{LV},D_{V},L,V)=E_{v∼\mathrm{pdata}(v)}[\log(D_{V}(v))]+E_{l∼\mathrm{pdata}(l)}[1-\log(D_{V}(G_{LV}(l)))]$$

where v is the input visible image, $G_{VL}\left(v\right)$ is the generated laser image, l is the input laser image, $G_{LV}\left(l\right)$ is the generated visible image, $E_{l\sim \mathrm{pdata}\left(l\right)}$ is the expectation of the input laser image, $\mathrm{pdata}\left(l\right)$ is the distribution of the laser image, $E_{v\sim \mathrm{pdata}\left(v\right)}$ is the expectation of the input visible image, $\mathrm{pdata}\left(v\right)$ is the distribution of the visible image. $D_{L}$ distinguishs between real laser images and generated laser samples, and the goal of the discriminator is to maximize this; therefore, when inputting the real laser image, it is hoped that the value of $\log\left(D_{L}\left(l\right)\right)$ is larger, and when inputting the generated laser image, a larger the $1-\log(D_{L}\left(G_{VL}\left(v\right)\right)$ value is expected. Similarly, for the discriminator $D_{V}$, when inputting a real visible image, it is hoped that the value of $\log\left(D_{V}\left(v\right)\right)$ is larger, and when inputting a generated visible image, it is hoped that the value of $1-\log(D_{V}\left(G_{LV}\left(l\right)\right)$ is larger.

CycleGAN introduces a cycle consistency loss function, and the image that is reconstructed after an input image passes through the two generators in sequence should be consistent with the image itself. In $G_{LV}\left(G_{VL}\left(v\right)\right)\approx v$ and $G_{VL}\left(G_{LV}\left(l\right)\right)\approx l$, the cyclic consistency loss is obtained by calculating the L1 norm distance between the input image and the reconstructed image.

(3) $$L_{\mathrm{cyc}}(G_{LV},G_{VL})=E_{v∼\mathrm{pdata}(v)}[{\left\|G_{LV}(G_{VL}(v))-v\right\|}_{1}]+E_{l∼\mathrm{pdata}(l)}[{\left\|G_{VL}(G_{LV}(l))-l\right\|}_{1}]$$

where $G_{LV}\left(G_{VL}\left(v\right)\right)$ is the reconstructed visible image, $G_{VL}\left(G_{LV}\left(l\right)\right)$ is the reconstructed laser image, and $\|\cdot {\|}_{1}$ is the L1 norm.

### 3.3. Experimental Results and Analysis

The hardware and software configurations used in the experiment are shown in Table 1.

Four classical models, CycleGAN, Pix2pix, U-GAN-IT and StarGAN were used for image translation experiments. Experimental data were the laser and visible face datasets in Section 2.

**Figure 8:**
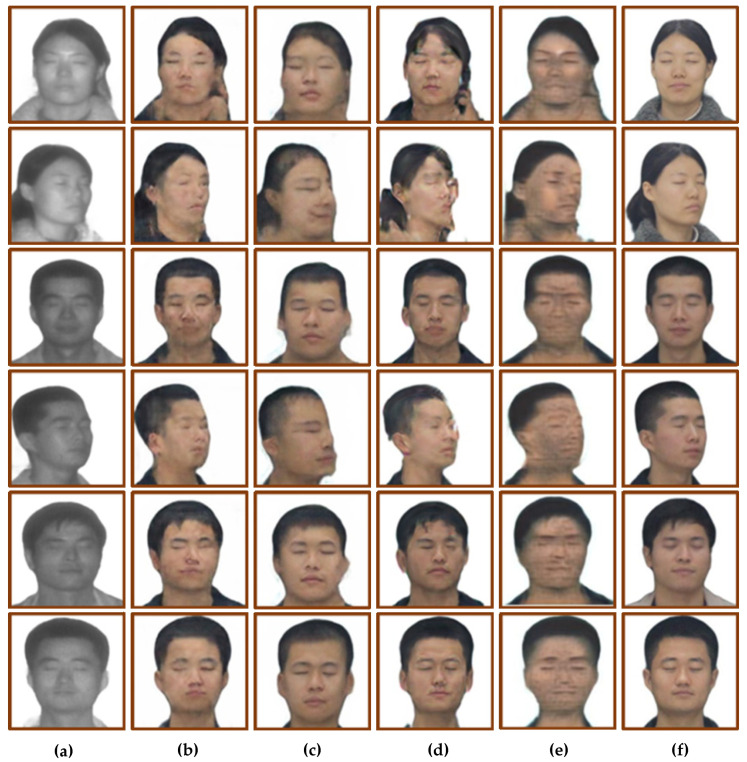
Four models generate face image results. (**a**) Input laser image. (**b**) CycleGAN. (**c**) Pix2pix. (**d**) U-GAN-IT. (**e**) StarGAN. (**f**) Ground truths.

The trained model was tested using the test set, and the results are shown in Figure 8. Figure 8a is the input laser image, Figure 8b is the face translation result of CycleGAN, Figure 8c is the face translation result of Pix2pix, Figure 8d is the face translation result of U-GAN-IT, Figure 8e is the face translation result of StarGAN, and Figure 8f is the real visible image. Subjective vision and objective quantification were used to analyze the translation results. At the same time, three evaluation indexes, including SSIM [18], PSNR and FID [19], were used to evaluate the four models. The quantitative results are shown in Table 2.

From the perspective of subjective vision, CycleGAN’s translation results show that the facial features are blurred and lack sufficient details, but the outline of the head is clear, and the translation of the boundaries between hair and face is relatively accurate. The facial contour of the translation result of Pix2pix is seriously distorted, and there are problems such as unclear contour. The translation results of U-GAN-IT have the phenomenon of facial features shifting, and some translation results have the texture of oil painting. The results of StarGAN cast a black shadow on the whole person, and the overall facial features are blurred, but the facial contours are relatively clear.

From the perspective of objective quantification, the SSIM values of CycleGAN and U-GAN-IT are close, both around 0.8, and the SSIM values of Pix2pix and StarGAN are close, both around 0.75. The PSNR values of CycleGAN, U-GAN-IT and StarGAN are close, and the PSNR value of Pix2pix is lower, about two times lower than the SSIM value of the first three models. The FID values of CycleGAN and Pix2pix are close, and the FID values of U-GAN-IT and StarGAN are relatively high, 60.97 and 32.15 higher than CycelGAN, respectively.

According to the above analysis from the two perspectives of subjective vision and objective quantification, CycleGAN has a good translation result. Therefore, using CycleGAN as the basic network, this paper improves CycleGAN to eliminate the problem of facial ambiguity in laser–visible-face translation using the CycleGAN model.

## 4. Improved CycleGAN

### 4.1. Improvement of the Adversarial Loss Objective Function

CycleGAN is not a face translation network, and may learn incorrect mappings during training, resulting in poor translation results. For example, with this problem, blurred facial features appear in the translated visible image.

The adversarial loss objective function in CycleGAN uses the cross-entropy loss function. The cross-entropy loss is mainly used for logical classification, pays too much attention to the accuracy of the prediction probability of the correct label during the training process, and ignores the difference in the incorrect label. This can lead to the saturation phenomenon, resulting in gradient dispersion. In addition, when the cross-entropy loss function is used as the objective function, when the discriminator discriminates the image as true, even if the image is far from the real data distribution, the generator will not optimize the image again. As a result, the generator learns an incorrect mapping; for example, the pictures generated by the generator are blurred. The objective function of the adversarial loss is changed from the cross-entropy loss function to the least-squares loss function [20]. When the discriminator discriminates the image as true, it will be re-discriminated because the image is far from the real data distribution.

Improved adversarial loss:

(4) $$L_{GAN}(G_{VL},D_{L},L,V)=E_{l∼\mathrm{pdata}(l)}[{\left(D_{L}(l)-1\right)}^{2}]+E_{v∼\mathrm{pdata}(v)}[D_{L}{\left(G_{VL}(v)\right)}^{2}]$$

(5) $$L_{GAN}(G_{LV},D_{V},L,V)=E_{v∼\mathrm{pdata}(v)}[{\left(D_{V}(v)-1\right)}^{2}]+E_{l∼\mathrm{pdata}(l)}[D_{V}{\left(G_{LV}(l)\right)}^{2}]$$

The ai of the least-squares method for the discriminator is to minimize the objective function, where $D_{L}\left(l\right)$ discriminates the authenticity of the laser image, and $D_{L}\left(G_{VL}\left(v\right)\right)$ discriminates the authenticity of the generated laser image. $D_{V}\left(v\right)$ discriminates the authenticity of the visible image, and $D_{V}\left(G_{LV}\left(l\right)\right)$ discriminates the authenticity of the generated visible image.

### 4.2. Add Identity Loss Function

**Figure 9:**
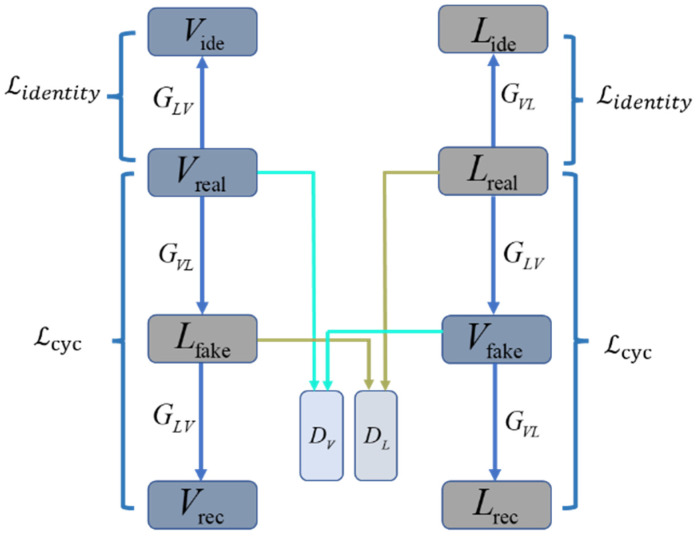
Modified network structure.

CycleGAN has a cycle consistency loss function based on cycle reconstruction, but the following situation is very easy to appear during training. After the previous generator on the same link learns the error map, the second generator also learns the error map, and the two error maps lead to a situation where the cycle consistency loss function is small but the generated picture is not effective. For example, in this experiment, blurry images and hairline mapping errors occur. To enhance the constraints on the generator and improve the quality of the generated images, the identity loss function is introduced, as shown in Figure 9. The reference identity loss [6] is used to enhance the hue accuracy; however, in face translation, the dataset used contains Asian faces, which have a single skin color and little color change. In this experiment, identity loss has no restrictions on skin color.

$G_{LV}$ has the ability to generate visible domain images, and the distribution of the generated images after feeding the image v into the $G_{LV}$ should be as close as possible to the distribution of the input image v. $G_{VL}$ has the ability to generate laser domain images, and the distribution of the generated images after feeding the images l into $G_{VL}$ should be as close as possible to the distribution of input image l. In training, the L1 norm distance between the input image v and the generated image $G_{LV}\left(v\right)$, and the L1 norm distance between the input image l and the generated image $G_{VL}\left(l\right),$ are calculated, adding the two results together to find the identity loss.

Identity loss is defined as:

(6) $$L_{identity}(G_{LV},G_{VL})=E_{v∼\mathrm{pdata}(v)}[{\left\|G_{LV}(v)-v\right\|}_{1}]+E_{l∼\mathrm{pdata}(l)}[{\left\|G_{VL}(l)-l\right\|}_{1}]$$

where $G_{LV}\left(v\right)$ represents the input of image v into generator $G_{LV}$, and $G_{VL}\left(l\right)$ represents the input of image l into generator $G_{VL}$. $\|G_{LV}\left(v\right)-v{\|}_{1}$ is used to calculate the L1 norm distance between $G_{LV}\left(v\right)$ and v, and $\|G_{VL}\left(l\right)-l{\|}_{1}$ is used to calculate the L1 norm distance between $G_{VL}\left(l\right)$ and l.

Total loss:

(7) $$\begin{array}{cc} L(G_{LV},G_{VL},D_{L},D_{V}) & =L_{GAN}(G_{LV},D_{V},L,V)+L_{GAN}(G_{VL},D_{L},L,V)\\ & +\lambda L_{cyc}(G_{LV},G_{VL})+\alpha L_{\mathrm{identity}}(G_{LV},G_{VL}) \end{array}$$

where λ and α are the weights of the cycle consistency loss function and the identity loss function, respectively.

### 4.3. Experimental Results and Analysis

Experimental data were the laser and visible face datasets in Section 2. The optimizer chose the Adam algorithm with parameters beta1 set to 0.5 and beta2 set to 0.999. The initial learning rate was 0.002, the learning rate of the first 100 epochs was 0.002, and the last 100 epochs were reduced at a rate of approximately 1% until reaching zero. λ was the weight parameter of cycle consistency loss, set to 10, α was the weight parameter of identity loss, set to 5.

**Figure 10:**
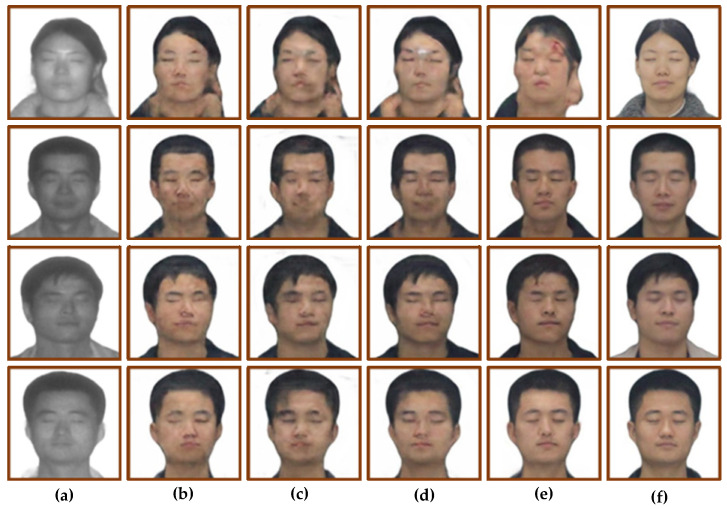
Ablation study: (**a**) Input face photos. (**b**) CycleGAN. (**c**) Add identity loss. (**d**) Use the least squares loss function. (**e**) Ours. (**f**) Ground truth.

The experimental comparison results are shown in Figure 10. Figure 10a shows the input laser image, Figure 10b shows the face translation results of CycleGAN, Figure 10c shows the face translation results, adding the identity loss function, and Figure 10d shows the face translation result of modifying the adversarial loss objective function. Figure 10e shows the face translation result of adding the identity loss function and modifying the adversarial loss objective function, and Figure 10f shows the real visible light image. Subjective vision and objective quantification were used to analyze the translation results. Using SSIM, PSNR, and FID as objective quantitative evaluation methods, the objective quantitative results are shown in Table 3.

From the perspective of subjective vision, the image translation results of Figure 10c,d have more obvious facial features than the image translation results of Figure 10b. However, both Figure 10c,d have a blurred hairline. Compared with the translation result of Figure 10b, the translation results of Figure 10e eliminate the problem of blurred images. At the same time, the facial features are relatively clear, with sufficient details, and the boundaries of hair and skin are clear. However, there is a little deviation in the mapping relationship of individual images, such as erythema on the forehead of the female image, and broken hair being translated into skin.

From the perspective of objective quantification, after adding the identity loss function to the CycleGAN model, the SSIM value and PSNR value are almost unchanged, but the FID value is reduced by 5.54. After the adversarial loss objective function for CycleGAN is modified to the least-square loss function, the SSIM value and PSNR value are almost unchanged, but the FID is reduced by 9.53. After adding the identity loss and improving the adversarial loss objective function to CycleGAN, the SSIM value of the improved model is similar, the PSNR is increased by 1, and the FID is decreased by 11.22.

**Figure 11:**
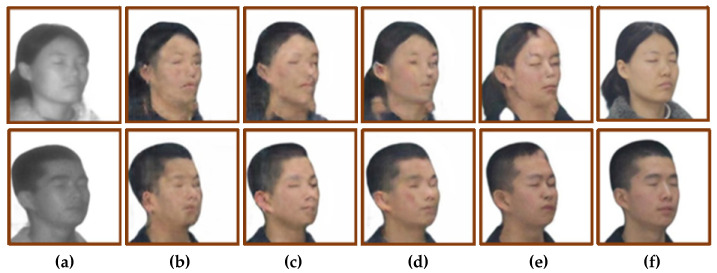
Ablation study of side face: (**a**) Input face photos. (**b**) CycleGAN. (**c**) Add identity loss. (**d**) Use the least squares loss function. (**e**) Ours. (**f**) Ground truth.

For the translation of frontal faces, the improved network achieves good results, but the modified network has flaws in the profile translation. For example, the dividing line between hair and face is mistranslated. The results of profile translation were analyzed from the two perspectives of subjective vision and objective quantification. Figure 11 shows the results of profile image translation, and Table 4 shows the objective quantification results of profile translation.

From the perspective of subjective vision, comparing the translation results of Figure 11b, the translation results of Figure 11c are also blurred, and the overall picture is not clear enough. The image translation results of Figure 11d are slightly better, eliminating the meaningless ripples in Figure 11b, but the facial features are also not clear enough. Compared with the translation results of Figure 11b, the translation results of Figure 11e eliminate the problem of blurred images. At the same time, the facial features are relatively clear and have sufficient details, but the boundaries between hair and skin are mapped mistakenly.

From the perspective of objective quantification, after adding the identity loss function to CycleGAN, the SSIM value and PSNR value are almost unchanged, but the FID value is reduced by 20.9. After the adversarial loss objective function for CycleGAN is modified to the least-squares loss function, the SSIM value and PSNR value are almost unchanged, but the FID is reduced by 40.51. After adding the identity loss and improving the adversarial loss objective function for CycleGAN, the SSIM value of the improved model is increased by 3.9%, the PSNR is increased by 19.8%, and the FID is decreased by 23.75.

In sum, the modified model is more suitable for the translation of laser frontal face images, and the translation effect of laser side images is poor, so the network needs to be improved again.

## 5. Profile Image Translation

### 5.1. Improved Feature Extraction Part

Although the problem of blurred facial features is solved, the modified CycleGAN learns an incorrect mapping during the translation of profile images, resulting in poor translation results for profile images. The brightness change in the laser image is unobvious and the features are fuzzy, especially the facial features and facial skin, which have no obvious dividing line. These aspects are very unfavorable for feature extraction. At the same time, CycleGAN is not sensitive to changes in the shape and position of the face in the image, and the profile has a certain angle change, which leads to deviations in the generated results. The generator has not learned enough feature information, and the feature extraction module of the generator is improved, offering generator have deeper network layers to improve the feature extraction ability of the network.

RRDB [21] replaces ResNet as the feature extraction module. Compared with ResNet, RRDB module has more network layers, and the feature extraction ability can be improved by increasing the network depth. However, with the increase in network depth, it is easy to cause the problem of gradient disappearance and gradient explosion. By introducing dense connections, RRDB makes full use of the output features of each layer and they become the input of the next layer, so that the input characteristic information and gradient information will not be lost during transmission.

**Figure 12:**
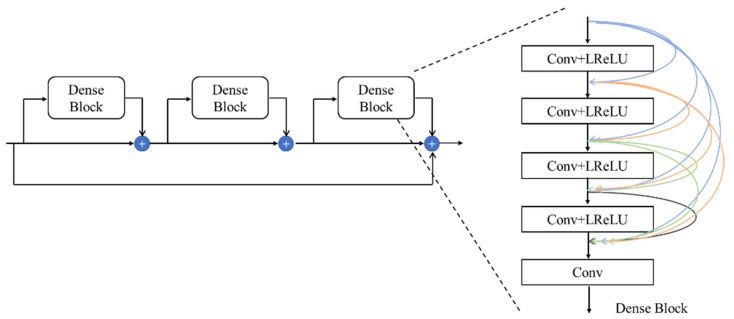
RRDB network structure.

RRDB includes three DenseBlocks; each DensBlock includes four Conv + LReLU structures and a convolutional layer, and dense connections are used between each layer. Compared with ResNet, RRDB has more network layers and can extract deeper feature information. Compared with ResNet’s Conv + BN + ReLU structure, DenseBlock uses Conv + LReLU structure and removes the batch normalization. The batch normalization will produce artifacts when the network is deep, so the batch normalization can be removed to make the network more stable and reduce the operation complexity. The RRDB network structure is shown in Figure 12.

### 5.2. Experimental Results and Analysis

**Figure 13:**
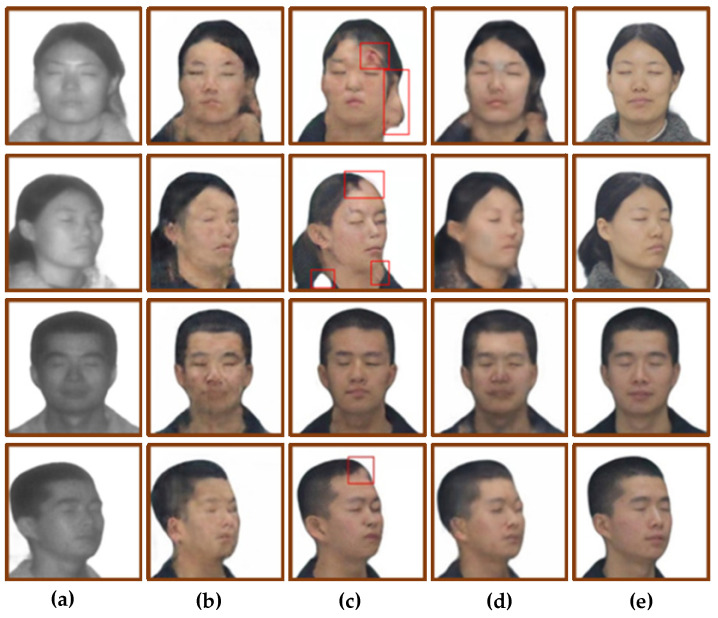
The translation results of changing the feature extraction module to RRDB. The red box shows the defects in the translated image of the first improved model. (**a**) Input laser image. (**b**) CycleGAN. (**c**) The first improved model. (**d**) Change to RRDB module. (**e**) Ground truth.

The modified model was used to train the laser-visible face dataset, and the test set was used to validate the model. The face translation results are shown in Figure 13. Figure 13a shows the input laser image, Figure 13b shows the translation result of CycleGAN, Figure 13c shows the translation result of the first improved model, Figure 13d shows the translation result of the second improved model, that is, the feature extraction module was changed to the RRDB module, and Figure 13e shows the real visible image. Using SSIM, PSNR, and FID as objective quantitative evaluation methods, the quantitative results are shown in Table 5.

Comparing the two improved models of CycleGAN, from the perspective of subjective vision, the second improvement led to a better translation effect on the side face. As shown in the red box in Figure 13c, there are errors in the translation of the dividing line between hair and facial skin, and the translation of broken hair into skin. On the basis of the first model improvement, the feature extraction part is improved, and the quality of the side face translation is effectively improved. By comparing the translation results of Figure 13c,d, the dividing line between hair and skin was clear, and there was no translation error for female broken hair.

From the perspective of objective quantification, the SSIM value of the translation results of the second improved model is 2.47% higher than that of the first improvement, and 3.75% higher than that of CycleGAN. The PSNR value of the translation results of the second improved model is similar to that of the first improved model, which is 1.02 higher than the value of CycleGAN. The FID value of the second improved translation results is 15.1 lower than the first improved model and 26.32 lower than CycleGAN.

In summary, the second improvement has a good effect on the translation of side-face images.

## 6. Conclusions

To solve the problem of poor translation of laser-visible face images, this paper proposes an improved CycleGAN network. The objective function of the original CycleGAN’s adversarial loss function is changed to the least-squares loss function, and the identity loss function is introduced, which reduces the image blurring and inconspicuous facial features in the image translation results. Focusing on the problem of wrong mapping in side face translation, a change from the feature extraction module of the generator to the RRDB module is proposed, which improves the feature extraction ability by increasing the network depth. The improved algorithm has better results in terms of both subjective vision and objective quantification. 

Through the research into deep learning and image translation, the laser–visible-face-image translation is completed, and the modal difference between laser-face image and visible-face image is eliminated, so that the laser image is transformed into a visible image, which is more suitable for human observation. This solves the problem of obtaining the target’s identity in surveillance videos under low-light conditions, so that the range-gated imaging equipment can be applied to the low-light environments at night, and make up for the problem that the visible imaging system cannot perform under low-light conditions. This means that the monitoring equipment can operate effectively all day long. 

## 7. Prospect

### 7.1. High-Resolution Image Translation

**Figure 14:**
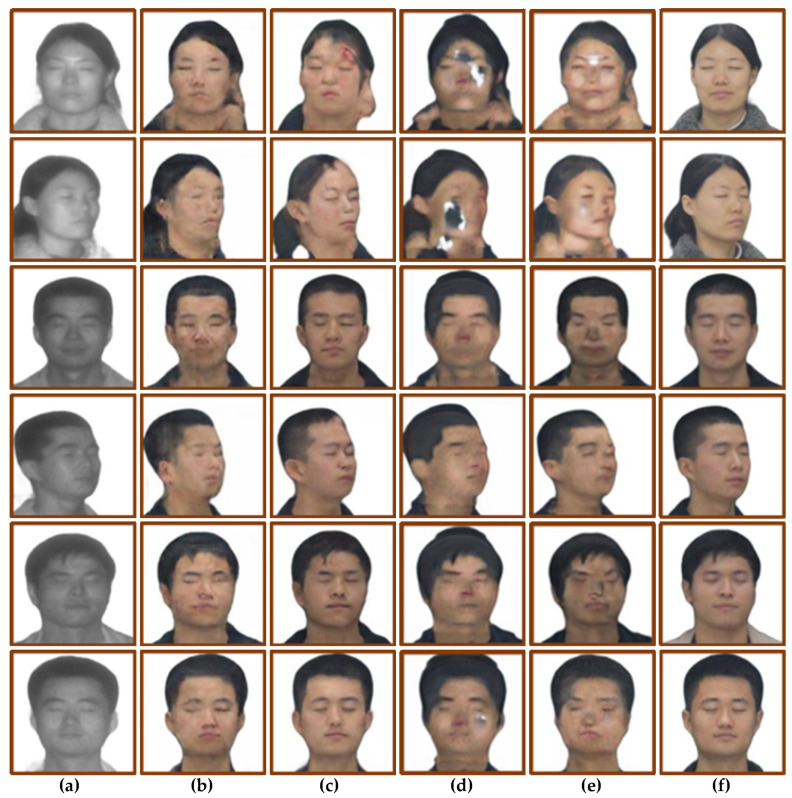
Image translation results with resolutions of 512 × 512 and 256 × 256. (**a**) Input laser image. (**b**) Image of 256 × 256 resolution using CycleGAN. (**c**) Image of 256 × 256 resolution using first improved CycleGAN. (**d**) Image of 512 × 512 resolution using CycleGAN. (**e**) Image of 512 × 512 resolution using first improved CycleGAN. (**f**) Ground truths.

In deep learning, the higher the image resolution, the more information and richer details it contains. To test the effect of high-resolution images on CycleGAN and the improved CycleGAN, the processed laser images and visible images are interpolated into images with a resolution of 512 × 512. The experimental results are shown in Figure 14 and Table 6.

From the perspective of subjective vision, when the images with a resolution of 512 × 512 were used as the training set, the quality of the translation results was not as good as when using images with a resolution of 256 × 256 as the training set. Compared with Figure 14b,c, the picture shown in Figure 14d has problems such as missing facial features, and the image shown in Figure 14e has the problem of blurred faces. From the perspective of objective quantification, when an image with a resolution of 512 × 512 is used as the training set, the PSNR and FID values are not as good as those of low-resolution images, and the SSIM is the opposite.

The laser image is interpolated to change the resolution from 256 × 256 to 512 × 512, and the features in the laser image become smooth and blurred, which is not conducive to feature extraction. In future work, the super-resolution method could be used to change the laser image from low-resolution to high-resolution, so that the image has more details and information, and the translation quality of the image is improved.

### 7.2. Improve Face Recognition Accuracy

In monitoring and security, most of the existing face datasets contain visible images, and it is difficult to directly recognize the laser face in the visible face dataset. It is necessary to translate laser-face images into visible-face image, and then perform face recognition. The FaceNet model can be used to test the facial recognition accuracy of images translated by the improved model. FaceNet [22] has trained model parameters and a complete backbone network, and the recognition model does not need to be improved. The facial recognition accuracy calculation process has three steps. First, the visible images in the training dataset are used as the face database, and the generated images are used as the faces to be recognized. The facial features of the database and generated images are extracted through FaceNet. Second, the Euclidean distance between the generated image and the face of the database is calculated through facial features. Third, according to the Euclidean distance, the facial recognition accuracy of rank-1, rank-5, and rank-10 are calculated, and the CMC curve is drawn.

**Figure 15:**
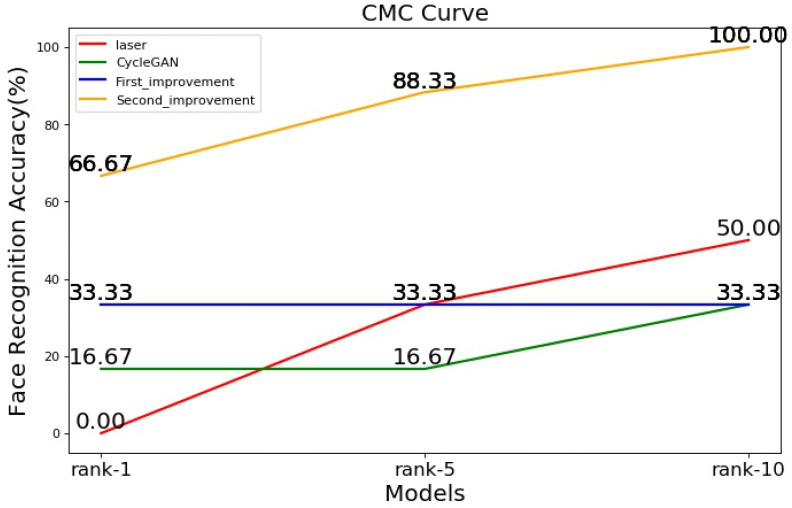
CMC curve of face recognition accuracy.

Figure 15 shows the facial recognition accuracies of rank-1, rank-5, and rank-10, with rank-1 being the highest precision. When rank-1, the facial recognition accuracy is highest, with an increased face-recognition efficiency. The facial recognition accuracy of the original laser image and the image translated by the three models generally increases with the decrease in precision, while the facial recognition accuracy of the second improved model is the highest in terms of the three precision ratings. When rank-10, the accuracy of the second improved model reached 100%, but when rank-1, the accuracy was only 66.67%, and the lower the precision, the higher the recognition accuracy. The face recognition accuracy of the second improvement was much higher than that of the first improvement, indicating that the second improved framework not only obtained a good result for the translation of the front face, but also improved the translation accuracy of the side face. Although the facial recognition accuracy of the second improved model was somewhat improved compared to the original laser image, the face recognition accuracy was lower at rank-1. This is because translated images differ from real images in terms of their facial recognition features.

Although the facial recognition accuracy of the second improved model improved compared to the facial recognition accuracy of the original laser image, the recognition accuracy did not meet expectations when rank-1. The next step will be to modify the network to improve the facial recognition accuracy.

### 7.3. Others

The current study focuses on the front face and the 30-degree side-face, but it is possible for the head to present any angle and posture in the monitoring equipment. How to improve the network’s extensiveness regarding face angle and posture is the focus of future research.

This paper mainly studies laser face translation, and the next step will be to explore the laser-visible image translation of more objects, such as human bodies, vehicles, etc. For example, at night, when the visible light device cannot image illegal vehicles, laser imaging can be used to obtain vehicle images, laser-visible image translation can be performed on the vehicle, and the body color and structure information can be obtained, which will help the police to quickly locate the illegal vehicle.

## Figures and Tables

**Table 1:** Configuration of hardware and software.

Hardware or Software	Technical Parameter
Operating system	Windows 10 Home Chinese
GPU	NVIDIAGTX-3090
CPU	Intel(R)Xeon(R)Silver4116
Memory	24 GB
Deep learning library	Pytorch 1.8.0
Programming language	Python 3.7.6

**Table 2:** Quantitative results of four models.

	Method	SSIM	PSNR	FID
Model				
CycleGAN		0.80	15.27	124.56
Pix2pix		0.75	13.55	126.19
U-GAN-IT		0.81	15.97	156.71
StarGAN		0.75	15.63	185.53

**Table 3:** Quantitative results of improved CycleGAN model.

	Method	SSIM	PSNR	FID
Model				
CycleGAN		0.80	15.27	124.56
Add identity loss		0.79	15.14	119.02
Least squares loss		0.81	15.85	115.03
Ours		0.81	16.27	113.34

**Table 4:** Quantitative results of improved CycleGAN for profile.

	Method	SSIM	PSNR	FID
Model				
CycleGAN		0.76	12.60	186.10
Add identity loss		0.76	12.67	165.12
Least squares loss		0.77	13.36	145.59
Ours		0.79	15.10	162.35

**Table 5:** Quantitative results of profile face translation.

	Method	SSIM	PSNR	FID
Model				
CycleGAN		0.80	15.27	124.56
First improvement		0.81	16.27	113.34
Second improvement		0.83	16.29	98.24

**Table 6:** Compare image translation quality at different resolutions.

	Method	SSIM	PSNR	FID
Model				
CycleGAN		* 0.80/0.82	* 15.27/13.23	* 124.56/184.47
First Improvement CycleGAN		* 0.83/0.84	* 16.29/14.85	* 98.24/128.36

* 256 × 256 resolution results /512 × 512 resolution results.

## Data Availability

Not applicable.
